# Fast analysis of scATAC-seq data using a predefined set of genomic regions

**DOI:** 10.12688/f1000research.22731.2

**Published:** 2020-05-28

**Authors:** Valentina Giansanti, Ming Tang, Davide Cittaro

**Affiliations:** 1 Department of Informatics, Systems and Communication, University of Milano-Bicocca, Milan, Italy; 2 Center for Omics Sciences, IRCCS San Raffaele Institute, Milan, Italy; 3 FAS informatics, Harvard University, Cambridge, MA, USA

**Keywords:** single cell, scATAC-seq, pseudoalignment

## Abstract

**Background:** Analysis of scATAC-seq data has been recently scaled to thousands of cells. While processing of other types of single cell data was boosted by the implementation of alignment-free techniques, pipelines available to process scATAC-seq data still require large computational resources. We propose here an approach based on pseudoalignment, which reduces the execution times and hardware needs at little cost for precision.

**Methods:** Public data for 10k PBMC were downloaded from 10x Genomics web site. Reads were aligned to various references derived from DNase I Hypersensitive Sites (DHS) using *kallisto *and quantified with *bustools*. We compared our results with the ones publicly available derived by *cellranger-atac*. We subsequently tested our approach on scATAC-seq data for K562 cell line.

**Results: **We found that *kallisto *does not introduce biases in quantification of known peaks; cells groups identified are consistent with the ones identified from standard method. We also found that cell identification is robust when analysis is performed using DHS-derived reference in place of *de novo* identification of ATAC peaks. Lastly, we found that our approach is suitable for reliable quantification of gene activity based on scATAC-seq signal, thus allows for efficient labelling of cell groups based on marker genes.

**Conclusions:** Analysis of scATAC-seq data by means of *kallisto *produces results in line with standard pipelines while being considerably faster; using a set of known DHS sites as reference does not affect the ability to characterize the cell populations.

## Introduction

Recent technological advances in single-cell technologies resulted in a tremendous increase in the throughput in a relatively short span of time ^1^. The increasing number of cells that could be analyzed prompted a better usage of computational resources. This has been especially true for the post-alignment and quantification phases. As a consequence, it is today feasible to run the analysis of single cell data on commodity hardware with limited resources ^2^, even when the number of observables is in the order of hundreds of thousands. Conversely, the analysis steps from raw sequences to count matrices lagged for some time. Alignment to the reference genome or transcriptome is largely dependent on classic aligners, without any specific option to handle single-cell data, with the notable exception of the latest implementation of STARsolo in the STAR aligner ^3^.

More recently, analysis of Next generation sequencing (NGS) data benefits from technologies based on *k*-mer processing, allowing alignment-free sequence comparison ^4^. Most of these technologies require a catalog of *k*-mers expected to be in the dataset and, hence, subject of quantification. RNA-seq analysis relies on the quantification of gene/transcript abundances and, while it is possible to perform *de novo* characterization of unknown species in every experiment, it is common practice ^5, 6^ to rely on a well-defined gene model such as GENCODE ^7^ to quantify expressed species. It is then possible to efficiently perform alignment-free analysis on transcripts to quantify gene abundances. Tools implementing this approach such as *kallisto* ^8^ or *salmon* ^9^ have been quickly adopted on a wide scale. Moreover, a recent implementation of *kallisto* extended its capabilities to the analysis of single cell RNA-seq data ^10^ by direct handling of cell barcodes and UMIs, allowing the analysis of such data in a streamlined way. Of notice, a scRNA-seq oriented implementation of *salmon* has been recently developed ^11^.

Analysis of epigenetic features by ATAC-seq requires the identification of enriched peaks along the genome sequence. This is typically achieved using peak callers such as MACS ^12^, with tuned parameters. Since ATAC-seq signal mirrors DNA accessibility as mapped by DNase-seq assays ^13^ and catalogs of DNase I Hypersensitive Sites (DHS) are available ^14, 15^, it should be possible to perform reference-based ATAC-seq analysis in a way much similar to what is performed for RNA-seq analysis. In this paper we show it is indeed possible to perform single-cell ATAC-seq analysis using *kallisto* and *bustools*, with minor tweaks, using an indexed reference of ~1 million known DHS sites on the human genome.

## Methods

### Single cell ATAC-seq data

Single cell ATAC-seq data for PBMC were downloaded from the 10x Genomics public datasets ( https://support.10xgenomics.com/single-cell-atac/datasets/1.1.0/atac_v1_pbmc_10k) and include sequences for 10k PBMC from a healthy donor. We used the Peak by cell matrix HDF5 (filtered) object as our ground truth.

Raw sequences for single cell ATAC-seq data for K562 cell line were downloaded from Short Read Archive (GEO ID GSE112200).

### Generation of *kallisto* index

We downloaded the DNase I Hypersensitive Sites (DHS) interval list for hg19 genome from the Regulatory Elements DB ^16^. Intervals closer than 500 *bp* were merged using bedtools ^17^.

We extracted DNA sequences for DHS intervals and indexed corresponding fasta files using *kallisto index* (v0.46.0) with default parameters, resulting in an index for the full DHS set (iDHSfull) and an index for the merged set (iDHS500). The same procedure was performed for the peak set identified by *cellranger-atac* and distributed along with the data (iMACS).

### Processing of Chromium 10x data

*kallisto* requires the definition of the unique molecular identifiers (UMI) and cellular barcodes (CB) in a specific fastq file. For standard Chromium scRNA-seq data, these are substrings of R1 and RNA is sequenced in R2. Chromium scATAC-seq reads are not structured in the same way: paired end genomic reads are in R1 and R3, R2 includes only the 16 *bp* cellular barcode. In addition, kallisto bus expects only a single read with genomic information. Therefore we simulated appropriate structures in three different ways:

- **1.** by adding 12 random nucleotides and mapping the R1 file (forward read):
kallisto bus -x 10xV2 modified_R1.fastq.gz 
pbmc_10k_R1.fastq.gz
- **2.** by extracting sequences of different length *n* (5, 10, 15, 20) from the 5' of R3 (reverse read) and mapping the R1 file:
kallisto bus -x 1,0,16:2,0,n:0,0,0 
pbmc_10k_R1.fastq.gz 
pbmc_10k_R2.fastq.gz 
pbmc_10k_R3.fastq.gz
- **3.** by extracting sequences of different length *n* (5, 10, 15, 20) from the 5' of R1 and then mapping the R3 file:
kallisto bus -x 1,0,16:2,0,n:0,0,0 
pbmc_10k_R3.fastq.gz 
pbmc_10k_R2.fastq.gz 
pbmc_10k_R1.fastq.gz

**Figure 1.**
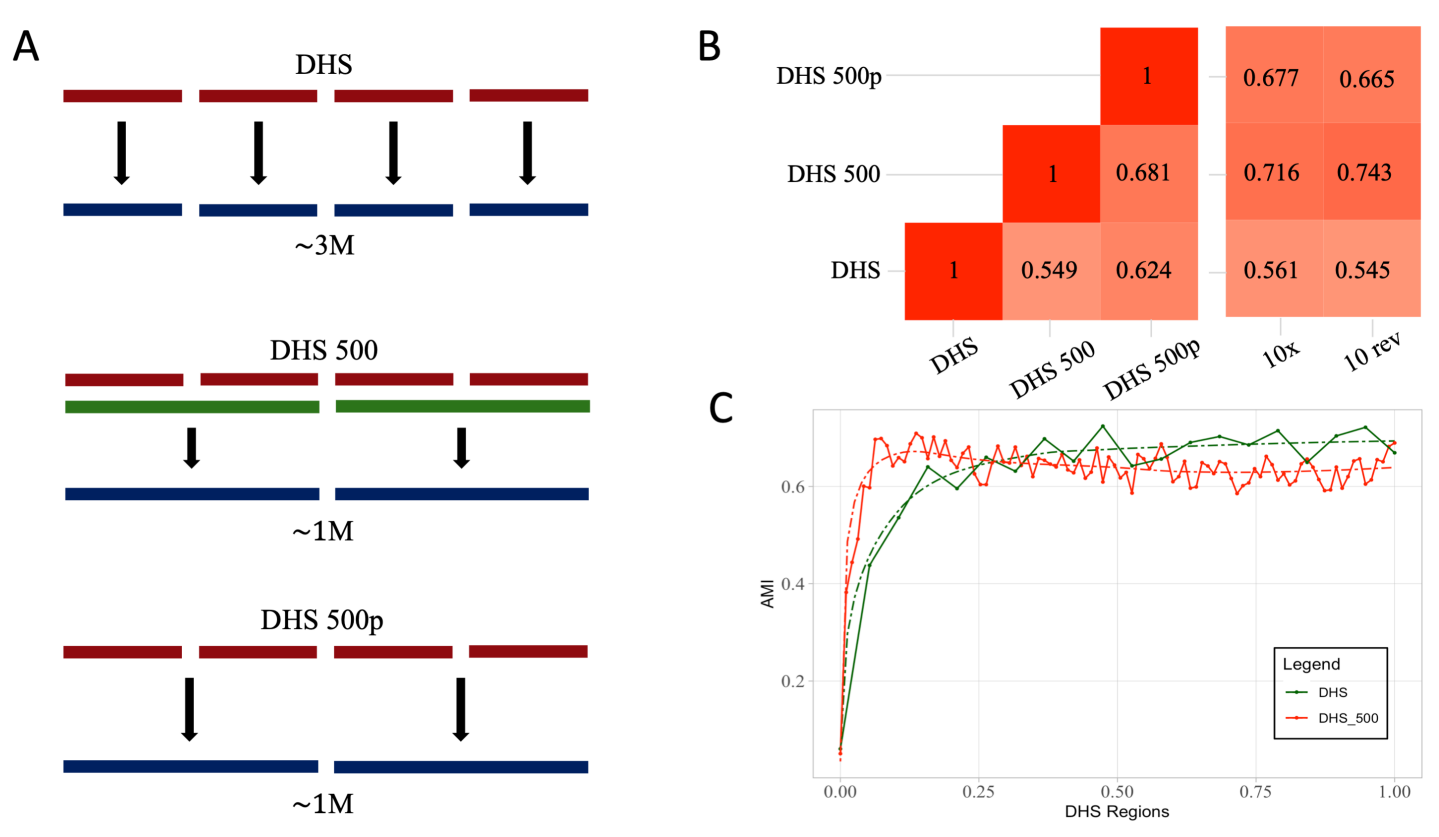
( **A**) Graphical depiction of processing of pseudoalignment over DHS, based on three DHS derived indices. The first (DHS) generated by *kallisto* on ~3 *M* DNase I sites, the second (DHS500) by merging regions closer than 500 *bp* and the last (DHS500p) by projecting the result of DHS index to DHS500 using bustools capabilities. ( **B**) Heatmaps representing MI scores for the DHS derived matrices. The heatmap on the left reports the pairwise MI values between DHS, DHS500 and DHS500p strategies. The heatmap on the right represents MI values comparing the DHS derived strategies to the *cellranger-atac* (10x) results or 10- *rev* strategy. DHS500 strategy achieves the highest scores. ( **C**) AMI values comparing DHS (green line) and DHS500 (red line) strategies to *cellranger-atac* at different thresholds on the number of regions considered in the analysis. When approximately 50,000 regions are included, the AMI stabilizes at its maximum. Dashed lines represent the fit curves.

We will refer to the second set of simulation as *n-fwd* and to the third set as *n-rev*, where *n* is the number of nucleotides considered as UMI. We also applied two different summarization strategies for *bustools count* step. In the first approach, pseudocounts are not summarized, the number of features matches the size of the index; in the second approach, summarized, we let *bustools map* counts on iDHSfull to the merged intervals ( Figure 1A).

### Processing of Fluidigm C1 data

Reads were aligned to reference genome (hg19) using bwa mem (v0.7.12) ^18^, deduplication was performed using samblaster (v0.1.21) ^19^. Only R2 were aligned in *bwa SE* configuration. Individual BAM files were merged using samtools and peaks were called from the pseudo-bulk using MACS2 (v2.2.7.1) ^12^ (paired end options: -q 0.1 --nomodel --shift 0, single read options: -q 0.1 --nomodel --shift -100 --extsize 200). Quantification was performed using bedtools multicov (-q 15).

*kallisto quant* was run with default parameters for paired end data. Only R2 were processed in *kallisto quant SE* with specific options (--single -l 300 -s 20). Individual counts from abundance files were merged using tximport ^20^.

In order to perform *kallisto bus* analysis we generated a set of 288 random CB which were used to create 288 matched fastq files. Once all read pairs and cellular barcodes have been concatenated into R1, R2 and CB fastq files, we ran *kallisto bus* with the same strategy used for PBMC data (-x 1,0,16:2,0,10:0,0,0).

### Analsyis of single-cell data

Counts matrices were analysed using Scanpy (v1.4.2) ^2^ with standard parameters. In PBMC data, we filtered out cells that had less than 200 regions and regions that were not at least in 10 cells. In K562 data we only excluded regions that were not shared by at least 20 cells. The count matrices were normalized and log transformed. The highly variable regions were selected and the subsetted matrices processed to finally clusterized the data with the Leiden algorithm ^21^, setting resolution parameter to 0.2. Marker peaks were selected using Wilcoxon rank-sum test. Adjusted Mutual Information (AMI) was used to evaluate the concordance between the 10x and matrices derived from kallisto.

Cellular barcodes were extracted using UMITools ^22^, setting the expected number of cells to 10,000.

The PBMC matrices derived from *kallisto* and *cellranger-atac* were also imported into Seurat V3 ^23^. Gene activity score was calculated using the CreateGeneActivityMatrix function or directly summarized by *kallisto*. The annotated 10 *k* PMBC scRNA-seq Seurat object was downloaded from the link available in their v3.1 ATAC-seq Integration Vignette ( https://satijalab.org/seurat/v3.1/atacseq_integration_vignette.html).

Cell labels from the scRNA-seq data were transferred to scATACseq data using TransferData function based on the gene activity score. All the analyses were carried out using standard parameters. Jaccard similarities were evaluated using the scclusteval (v0.1.1) package ^24^.

## Results

### Limitations of *kallisto*-based analysis

At time of writing, *kallisto* does not natively support scATAC-seq analysis, though it can be applied to any scRNA-seq technology which supports CB and UMI. According to the *kallisto* manual, the technology needs to be specified with a tuple of indices indicating the read number, the start position and the end position of the CB, the UMI and the sequence respectively. In this sense, the technology specifier for standard 10x scRNA-seq with v2 chemistry is 0,0,16:0,16,26:1,0,0 (see *kallisto* manual for details). Using this logic, a single fastq file contains sequence information and UMI is always required. scATAC-seq from 10x genomics is sequenced in paired-end mode and there is no definition of UMI, reads are deduplicated after genome alignment.

*kallisto* requires an index of predefined sequences to perform pseudoalignment, typically transcript. When applied to scATAC-seq analysis, it does not allow for any epigenomic analysis, including the identification and quantification of enriched regions. Therefore, we computed an index on the genomic sequences for the 80,234 peaks identified by *cellranger-atac* and distributed along with fastq files. This ensures that the subsequent analysis were performed on the same regions and allowed us to quantify the bias, if any, introduced by *kallisto*.

### *kallisto* primary analysis on PBMC data

We tested different strategies to overcome the technical limits and the absence of UMI. We evaluated concordance of different approaches using AMI between cell groups identified with equal processing parameters. Analysis based on *cellranger-atac* results is considered as ground truth. Results are reported in Table 1.

**Table 1.** Comparision of *cellranger-atac* and *kallisto* analysis. The table reports Adjusted Mutual Information between single cell cluster assignments on *cellranger-atac* data and *kallisto* analysis. Different strategies to evaluate pseudoUMI are reported. All simulations raised high AMI values, both in the forward and reverse approach, except for the pseudoUMI of length 5. The 10-Reverse configuration reached the highest score.

Comparison	Forward	Reverse
10x vs 5nt	0.1854	0.1733
10x vs 10nt	0.7434	0.7625
10x vs 15nt	0.7571	0.7398
10x vs 20nt	0.7356	0.7520
10x vs Random	0.7272	*None*

We tested two main strategies: first, the R1 is pseudoaligned and the initial nucleotides of R2, cut at different thresholds, are used as UMI (pseudoUMI hereafter). As UMI is needed for deduplication, we reasoned that a duplicate in scATAC-seq should be identified by the same nucleotides in the first portion of the read, where quality is higher. We observed generally high values of AMI, with the notable exception of pseudoUMI 5 *nt* long. Since basecall qualities are generally higher for R1 and *kallisto* does not use qualities in pseudoalignment, we tested the strategy where R2 is mapped and R1 is used to derive pseudoUMI. Again, 5 *nt* pseudoUMI raised the worst results, while AMI values were slightly higher than the forward configuration. In particular, we noticed the highest AMI values when R2 is used and pseudoUMI is 10 *nt* long ( *AMI* = 0.7625). Second, we tested a configuration using R1 as sequence and 10 *nt* UMI randomly generated. Interestingly, concordance remains in line with previous experiments ( *AMI* = 0.7272).

These data indicate that *kallisto* is able to properly quantify enrichments in scATAC-seq and does not introduce a considerable bias.

### Analysis of DHS as reference

One major limitation of a *kallisto*-based approach to scATAC-seq is the lack of peak calling routines and the need of an index of sequences for pseudoalignments. Hence, we reasoned that we could use any collection of regions that putatively would be target of ATAC-seq experiments. Since ATAC-seq is largely overlapping DHS, we exploited regions defined in the ENCODE project ^25^. The DHS data provided by ENCODE includes 2,888,417 sites. We generated an additional dataset by merging regions closer than 500 *bp* into 1,040,226 sites. We performed pseudoalignment on the full dataset, on the merged dataset and on the full dataset summarized by *bustools* ( Figure 1A, see *Methods*). Pairwise comparison between performances of the three methods reveals lower values of AMI ( Figure 1B). Comparison with 10x data and the configuration 10 *-rev* previously performed shows high values of AMI when considering merged DHS intervals ( *AMI* = 0.7164 and 0.743 respectively). When pseudoalignmets are performed on the full DHS set, performance degrades to lower AMI values. Since the number of DHS intervals is considerably higher than the typical number of regions identifiable by ATAC-seq, we tested the trend of AMI at different cutoffs on the number of DHS included in the analysis ( Figure 1C). AMI reaches a plateau when approximately 50,000 regions are included into the analysis. This defines a reasonable target for filtering during preprocessing stages of scATAC-seq data. Taken together, these findings support the suitability of using *kallisto* for identification of cell identities in scATAC-seq without any prior knowledge of the epigenetic status of single cells.

### Identification of marker regions

**Figure 2.**
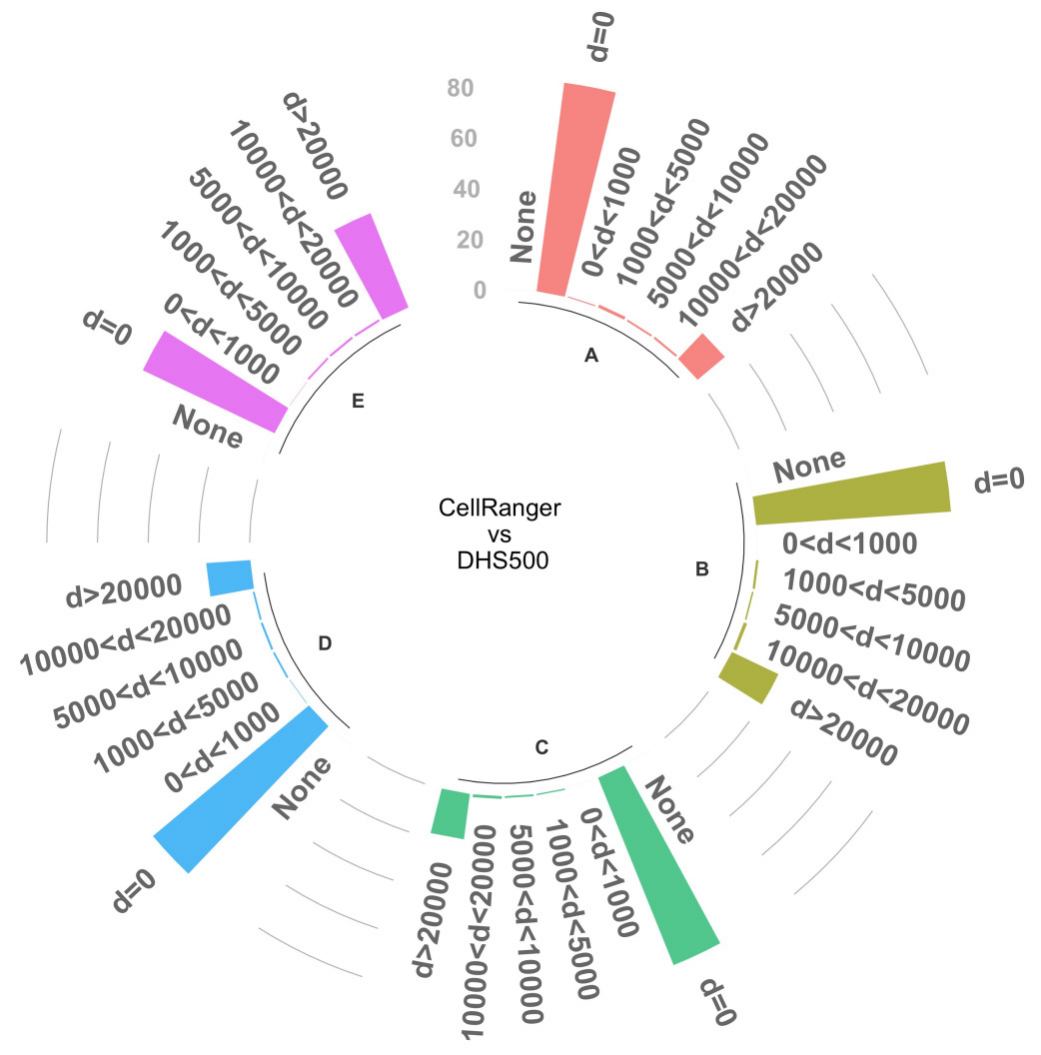
Analysis of peak concordance. The bars represent the proportion of marker peaks that are in common between DHS500 and *cellranger-atac*-based strategies at different distance thresholds. Only the top 1,000 significant peaks ( *p* < 0.05) were included in the analysis; the graph reports results for the 5 cell clusters ( **A**– **E**) that contain the required amount of significant markers. The chart also reports the proportion of peaks without any match ( *None*).

A crucial step in the analysis of scATAC-seq data is the identification of marker peaks which can be used to functionally characterize different clusters. We tested the ability of our reference-based approach to identify differential DNase I hypersensitive sites that are overlapping or close to peaks identified with standard analysis. To this end, we first matched cell groups from DHS500 to groups identified after *cellranger-atac*. We selected the top 1,000 peaks marking each DHS500 group and evaluated the concordance by mutual distance to the top 1,000 significant markers in the matched groups ( *p <* 0.05), we could identify significant markers only in five matched clusters. We found that the large majority of peaks ( *>*= 80%) were overlapping between the two strategies or closer than 20 *kb* ( Figure 2). These results confirm the substantial equivalence between the standard strategy and the reference-based one.

### Integration with scRNA-Seq data and cluster labeling

**Figure 3.**
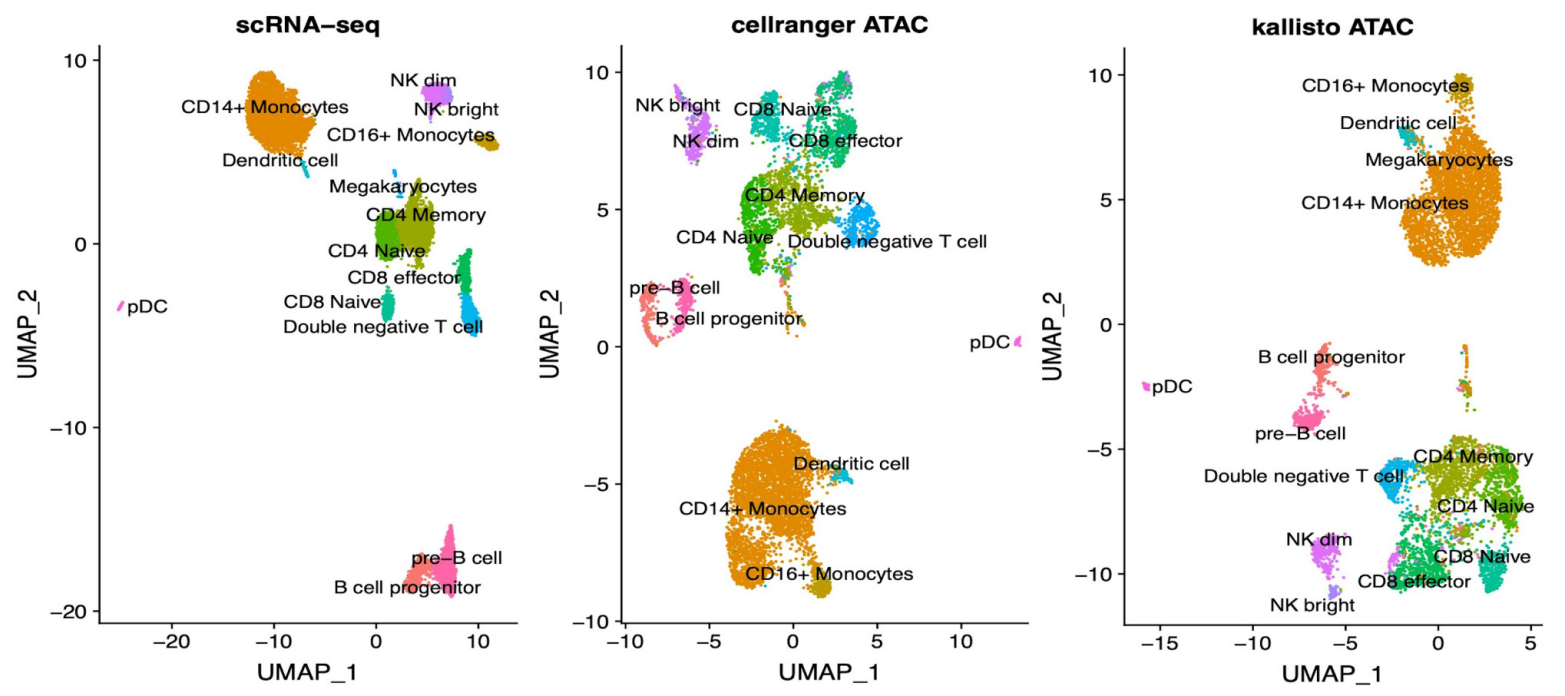
Results of label transfer from reference populations. The UMAP plot on the left represents scRNA-seq data of 10k PBMC as returned by Seurat vignette. The UMAP plots in the middle and on the right represent scATAC-seq analysis on *cellranger-atac* or *kallisto* analysis respectively. Cell clusters are consistent in their topology in the three plots, indicating the validity of *kallisto* for this kind of analysis.

**Figure 4.**
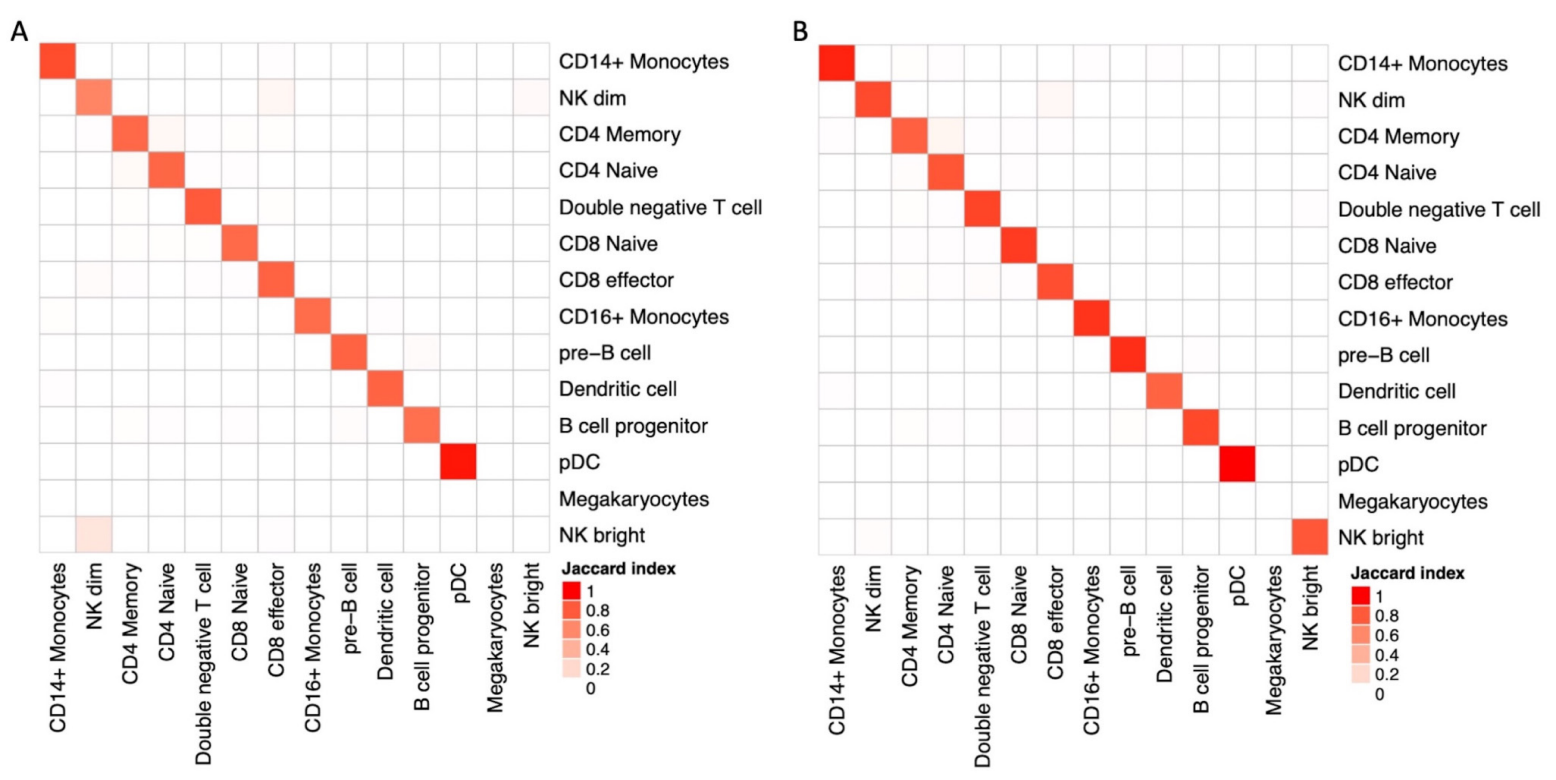
Analysis of Gene Activity Scores. ( **A**) Pairwise Jaccard similarity between cell annotations as a result of label transfer from RNA-seq data using Gene Activity Score evaluated by Seurat. Concordance between results after *cellranger-atac* (rows) and DHS500 (columns) are largely comparable, with the notable exception of NK subpopulations. ( **B**) Pairwise Jaccard similarity between cell annotations on DHS500 when Gene Activity Score is computed by Seurat (rows) or by *bustools* summarization step (columns).

In addition to the analysis of technical suitability of *kallisto* for the analysis of scATAC-seq data, we investigated its validity in extracting biological insight. To this end, we performed a more detailed analysis of PBMC data by label transferring using Seurat V3 ^23^, with the hypothesis that different approaches could lead to mislabeling of cells clusters. Matching is performed with the help of Gene Activity Scores calculated as sum of scATAC-seq counts over gene bodies extended 2 *kb* upstream the TSS, Seurat’s default approach. We applied the same transferring protocol on data derived from *cellranger-atac* counts and from the DHS500 approach ( Figure 3), finding no relevant differences in the UMAP embeddings. A detailed quantification of cluster matches reveals a slight deviance in the characterization of NK subpopulations ( Figure 4A). In addition to scores calculated by Seurat, we tested the ability of *bustools* summarization step to project and sum scATAC-seq values into Gene Activity using the identical mapping to extended gene bodies. We found that gene activity score obtained by kallisto is similar to Seurat’s CreateGeneActivityMatrix ( Figure 4B) in terms of cell labeling, with the additional advantage of reduced run time.

### Analysis of K562 cell lines

PBMC mixtures among the *de facto* standards in single cell benchmarks; it may be argued that the heterogeneity of the mixture can be easily revealed, implying that the differences between cell populations are large enough to be spotted also with suboptimal approaches. We analyzed scATAC-seq data for 288 K562 cells ^26^, profiled on a Fluidigm C1 apparatus, to test the consistency of our approach on a supposedly homogeneous population. Since sequences are available for each cell separately, we could extend our tests to the standard *kallisto* quantification procedure ( *kallisto quant*), performing separated cell-based pseudoalignments. We explored seven different strategies, either based on paired-end reads ( *bwa PE + MACS*, *bwa PE + DHS, kallisto quant PE*) or single reads ( *bwa SE + MACS, bwa SE + DHS, kallisto bus* and *kallisto quant SE*). We tested single read modality to accomplish a fair comparisions with *kallisto bus.* In our tests, *bwa PE + MACS* resembles a typical approach for the analysis of such data (as in 26). Strategies based on *kallisto* and strategies named with *DHS* make use of the DHS500 set of regions.

**Figure 5.**
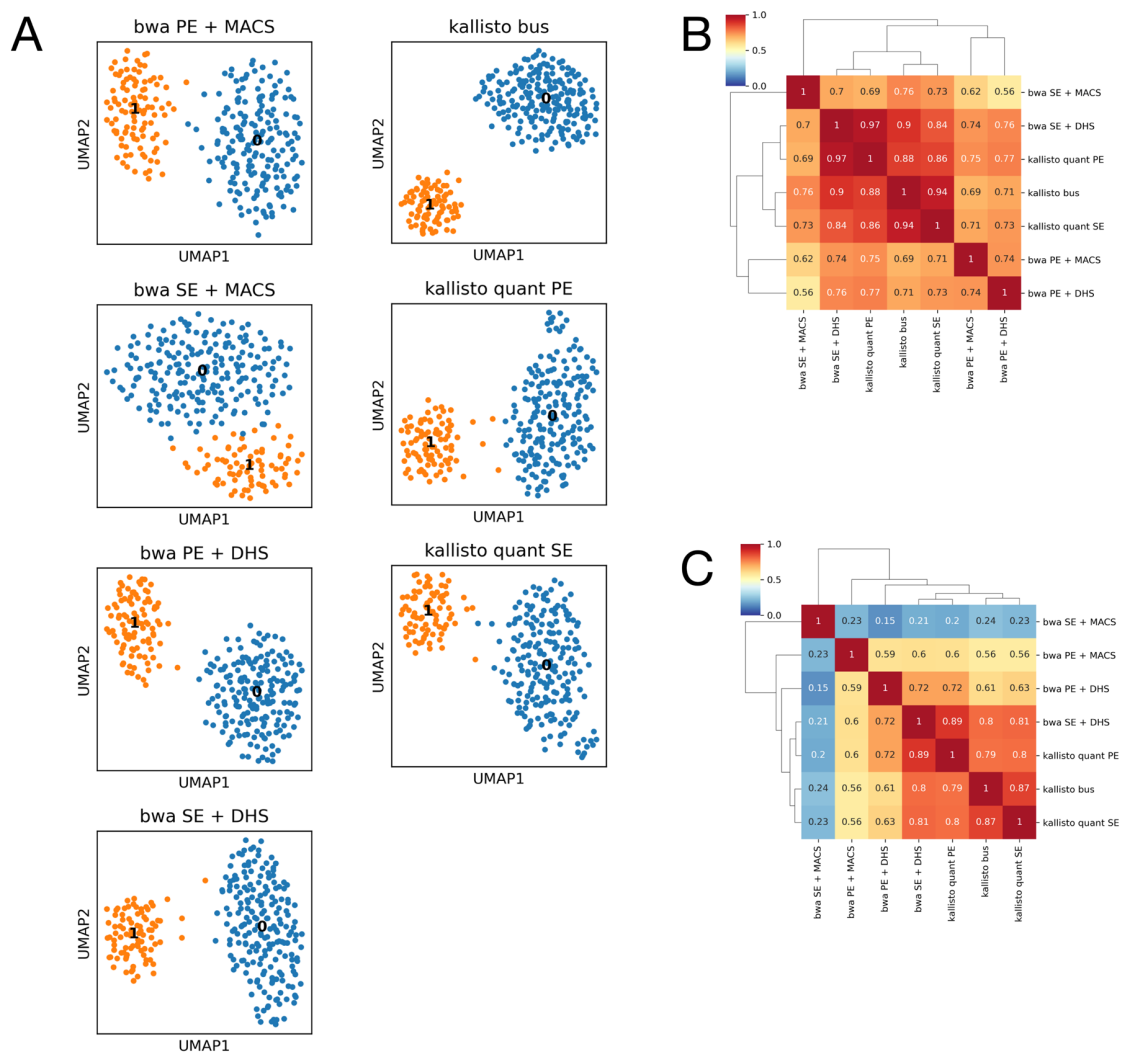
Analysis of K562 cell lines. Comparison of multiple standard- and reference-based approaches on scATAC-seq of K562 cell line. ( **A**) UMAP embeddings for the multiple approaches described in the text. All cases identify two major subpopulations. ( **B**) Pairwise Adjusted Mutual Information between all the approaches described in the main text. High AMI values indicate that all the approaches are consistent in identifying cell propertis. ( **C**) Pairwise Jaccard’s coefficients between marker peaks identified in each analysis. All approaches are able to identify a similar set of regions marking cell groups, with the exception of *bwa SE + MACS* which relies on a larger set of spurious regions.

Overall, we found a high concordance among all strategies. Two major cell groups could be identified using the equal processing parameters ( Figure 5A) and cells were found generally classified into consistent groups ( Figure 5B), with the notable exception of *bwa SE + MACS*. Excluding the latter, AMI ranges between 0.69 and 0.97. Interestingly, the comparison between *bwa PE + MACS* and *bwa PE + DHS* ( *AMI*=0.74) suggests that the major source of differences is the set of regions, not the alignment and quantification method. The concordance between marker regions, measured by Jaccard’s coefficient, reveals a similar configuration, again with the notable exception of *bwa SE + MACS* ( Figure 1C). This last approach is possibly biased by spurious ATAC peaks identified when only single reads are used: in this case MACS2 identified 17,125 peaks (average score 46.079), while in paired end configuration it identified 5,120 peaks (average score 65.919). Peaks shared by both the analyses have high quality (average score 86.104) while peaks specific of peaks identified after *bwa SE + MACS* are indeed low quality (average score 31.039). These findings indicate that single read mode is not suitable for *de novo* identification of ATAC peaks.

In all, analysis on less heterogeneous data confirm the suitability of *kallisto-*based and, more in general, reference-based approaches for the analysis of scATAC-seq experiments. **

### Computational resources

**Figure 6.**
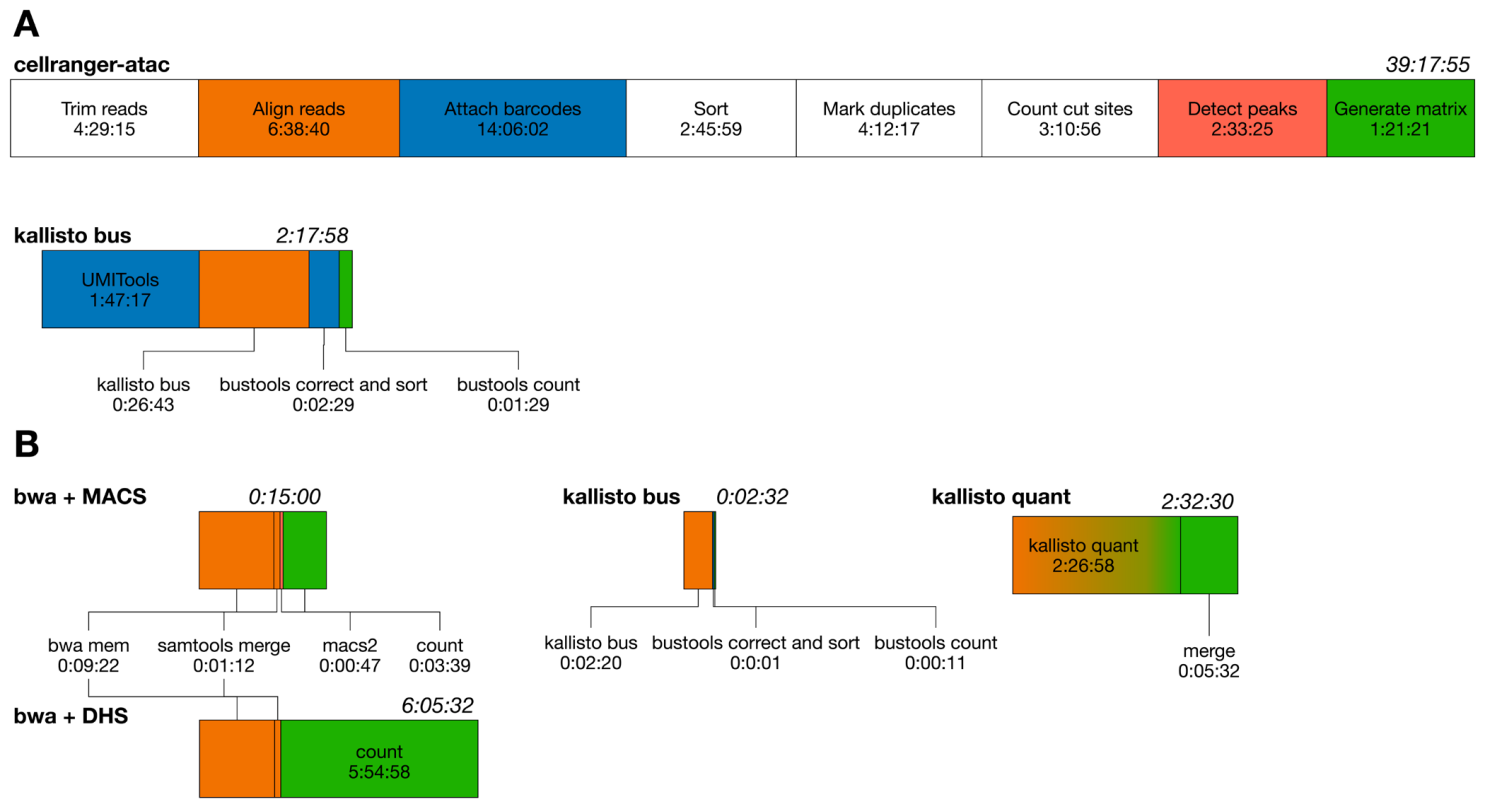
Runtime analysis. Graphical representation of runtimes for the datasets processed in this paper. Each box represents a separate step in a pipeline, box size is proportional to runtime in logarithmic scale. Colors in each box maps logically equivalent steps mirrored in different pipelines. ( **A**) Runtimes of *cellranger-atac* and *kallisto bus* on the PBMC 10k dataset. White boxes indicate steps that are not mirrored in both the analysis. ( **B**) Runtimes of all the approaches used in the analysis of K562 data. The gradient in *kallisto quant* indicates a hybrid step, which performs mapping and quantification. *bwa SE* pipelines have been excluded from the chart.

One of the most obvious advantages in using *kallisto* in place of alignment-based methods is the reduction of resources required to process raw sequences into a count matrix. We compared runtimes of the various approaches used through this work. First, we compared *cellranger-atac* pipeline and *kallisto* on a machine equipped with 12 CPU (Intel X5650@2.67GHz) and 72 Gb RAM using the PBMC dataset. While it took 46:49:57 hours for *cellranger-atac* to complete the analysis, its total runtime includes several post-processing and analysis steps. To make a fair comparison, we focused on pipeline steps that are mirrored in both the approaches (alignment, barcode assignment and counting) and the steps that are prerequisites to them ( Figure 6A). To this end, we also considered in the *kallisto* runtime an external application to identify valid cellular barcodes ( *UMITools*). This step can be replaced by any tool capable to return a list of valid cellular barcodes. The total effective time of *kallisto* is approximately 17x shorter, also because many processing steps are not required (initial trimming and BAM processing) or missing by design (peak calling). Our results are consistent with previous estimates on scRNA-seq data ^27^. In addition to reduced runtimes and pipeline simplicity, usage of *kallisto* implies reduced disk usage (12 Gb vs 40 Gb).

Analysis of the K562 datasets show reduced runtimes due to the smaller number of cells and sequences. Comparisons have been performed on the same 12 CPU platform, running 3 cells in parallel, 4 threads each, for *kallisto quant* and *bwa-*based pipelines. Coherently with the PBMC dataset, *kallisto bus* analysis is approximately 7x shorter than the default approach ( Figure 6B). Note, however, that raw sequences are generated for separate cells: alignment could be performed on as many computing units as the number of cells themselves. As an example, one could run 288 parallel alignments, reducing the total alignment step by a factor 96x (5.8s), assuming no impact on the I/O subsystem. The quantification step of *bwa*-based approach is impacted by the size of the peak list, which was three orders of magnitude smaller for *bwa PE + MACS* (5,120). A special case is the *kallisto quant* approach: we found the pseudoalignment step being much slower than the *bwa* counterpart. By looking at execution logs, we noticed that *kallisto* spends a large time in loading the reference in memory, this is repeated for each cell separately. *kallisto bus* loads the reference one time only, with beneficial impact on speed. As for disk usage, *kallisto bus* requires less space than *bwa PE + MACS* (393 Mb vs 1.2 Gb), while *kallisto quant* needs considerably more space (14 Gb), due to the ‘abundance.tsv’ text files produced by default during processing.

Lastly, it should be noticed that *kallisto* memory requirements in building the index are proportional to the number of *k*-mers found. The DHS500 database is composed by 682,100,489 *k*-mers and RAM allocation peaks at 37 Gb during indexing. The process itself takes 37.5 hours to complete.

## Discussion/conclusions

Analysis of differential chromatin properties, through ATAC-seq and other quantitative approaches, relies on the identification of peaks or enriched regions, It is often achieved with the same statistical framework used in analysis of differential gene expression ^28, 29^. Identification of peaks is a key difference between the two approaches. *De novo* discovery of unannotated transcripts has been shown to be possible in early times of NGS ^30^, but the large majority of analysis is performed on gene models. Conversely, analysis of epigenomes involves identification of regions of interest, although a large catalogues of such regions have been provided by several projects, such as the ENCODE project ^31^, the BluePrint project ^32^ or the GeneHancer database ^33^. In single cell analysis, for both scRNA-seq and scATAC-seq, identification of novel features may be an issue, especially because of the low coverage at which single cells are profiled. To our knowledge, this work is the first to test the feasibility of a reference-based approach to ATAC-seq analysis, with a special focus on single cell ATAC-seq. In combination, we tested the suitability of *kallisto* to quantify scATAC-seq, which maximizes the performances of the whole process. Our results suggest that identification of cell groups using a reference-based approach is not different from a standard pipeline. Not only cells could be classified in a nearly identical way, but also differential features are largely matched between the analysis. The most obvious advantage is the gain in speed and efficiency: once reads have been demultiplexed, *kallisto* analysis requires short execution times, in the order of minutes, with limited hardware resources. This advantage has been known for a while and, in fact, it has been demonstrated that it can be used on Rock64 hardware ^34^. We also anticipate that adoption of a reference-based strategy comes with additional advantages: in particular, functional annotations and gene associations are available for known regulatory regions ^25^ and, more recently, for DNase I Hypersensitive Sites ^15^. In the analysis of K562 cells, we highlighted a degradation of performances when a spurious region list is used, in our case peaks identified by MACS using single reads only. While best practices for ATAC-seq analysis are available ^35^, adoption of a reference-based approach could improve stability of results and their reproducibility.

Of course, our strategy has limitations that come from the unavailability of read positioning on the genome: in addition to the impossibility of identifying novel peaks, it is not possible to perform some ATAC-specific analysis, such as nucleosome positioning or footprinting of transcription factors in accessible regions. Indeed, these two can be overcome if standard alignment is used in place of pseudoalignment. Another limitation is the large amount of memory needed to index the DHS reference. Although indexing cannot be performed on less performing hardware, prebuilt indexes can be distributed as it is currently done for many aligners. As concluding remark we would like to underline that, although we showed that *kallisto* can be effectively used for analysis of scATAC-seq data, we are aware that it has not been conceived for that purposes; its interface needs some tweaks to work. For this reason, we advocate the development of tools which support scATAC-seq natively and other tools for postprocessing and data visualization.

## Data availability

### Source data

Single cell ATAC-seq data for 10k PBMCs dataset were downloaded from the 10x Genomics public datasets ( https://support.10xgenomics.com/single-cell-atac/datasets/1.1.0/atac_v1_pbmc_10k). Access to the data is free but requires registration. Raw sequences for K562 cells were downloaded from the Gene Expression Omnibus under the accession ID GSE112200 ( https://www.ncbi.nlm.nih.gov/geo/query/acc.cgi?acc=GSE112200).

### Extended data

Zenodo: vgiansanti/Kallisto-scATAC v1.1. https://doi.org/10.5281/zenodo.3834767 ^36^.

This project contains a detailed explanation of the procedures described in this work and the list of DHS sites; this is also available at https://github.com/vgiansanti/Kallisto-scATAC.

Extended data are available under the terms of the Creative Commons Attribution 4.0 International license (CC- BY 4.0).
