# Supplementary material for: Fast analysis of scATAC-seq data using a predefined set of genomic regions
Source: F1000Res. 2020 May 28;9:199. Originally published 2020 Mar 20. [Version 2] doi: 10.12688/f1000research.22731.2 (PMC7308914; doi:10.12688/f1000research.22731.2)
Supplement: Peer review history

# Peer review: Reviewer response for version 2

**DOI:** 10.5256/f1000research.26547.r64049

**Published:** 2020-06-15

**Authors:** Qiangfeng Cliff Zhang

**Affiliations:** 1 MOE Key Laboratory of Bioinformatics, Beijing Advanced Innovation Center for Structural Biology, Center for Synthetic and Systems Biology, School of Life Sciences, Tsinghua University, Beijing, China

I am happy that all my concerns are addressed and I like the method.

Is the rationale for developing the new method (or application) clearly explained?

Yes

Is the description of the method technically sound?

Yes

Are the conclusions about the method and its performance adequately supported by the findings presented in the article?

Yes

If any results are presented, are all the source data underlying the results available to ensure full reproducibility?

Yes

Are sufficient details provided to allow replication of the method development and its use by others?

Yes

Reviewer Expertise:

Bioinformatics, genomics, RNA structure, Genome structure, AI algorithms in biomedicine.

I confirm that I have read this submission and believe that I have an appropriate level of expertise to confirm that it is of an acceptable scientific standard.

# Peer review: Reviewer response for version 1

**DOI:** 10.5256/f1000research.25099.r62150

**Published:** 2020-05-06

**Authors:** Qiangfeng Cliff Zhang

**Affiliations:** 1 MOE Key Laboratory of Bioinformatics, Beijing Advanced Innovation Center for Structural Biology, Center for Synthetic and Systems Biology, School of Life Sciences, Tsinghua University, Beijing, China

Comments:

 The work by Giansanti *et al*. presents a novel and smart idea for scATAC-seq data analysis. It demonstrates the possibility of using a reference-based, pseudo-alignment method to reduce the computational requirement for scATAC-seq data analysis, with only a little sacrifice on precision. The idea is inspired by the using of pesudoalignment for bulk and single-cell RNA-seq quantification. Here they showed that with some tweaking of the input sequencing reads, they could use kallisto to analyze scATAC-seq data on a pre-defined set of DNase hypersensitive sites. They compared their results with the standard protocol (e.g. *cellranger-atac*) for peak quantification, single cell clustering, marker peaks identification, and gene activity score calculation.

 The results very nicely revealed the consistency on peak quantification between *kallisto*-based method and *cellranger-atac*. The cell clusterings were almost identical between the new reference-based method and canonical mapping strategy. And the gene activity scores by two different methods also agreed well with each other. The approach presented in this study thus could be a very efficient way for scATAC-seq data analysis.

 The following are a few comments/questions:

1. The method was only tested with one dataset - PMBC. In fact, single cell ATAC-seq data is usually very sparse. The PMBC dataset used in this study is of relatively high quality. The method remains to be tested on more datasets, especially on those of more sparse, lower-quality.
2. The key advantage of the method is presumably the much improved computational efficiency – there may be other advantages brought by the reference-based method. However, there is no results/statistics on the running time and memory usage in the manuscript. From the description, the improvement should be dramatic. I think it would be very nice to include a table or a figure to demonstrate the increase of computational efficiency. This could be a very helpful way to convince potential users. 
3. As in the above, this whole strategy is so different. It is thus possible for the method to be used for some other scATAC-seq data analysis with advantages not only in computational efficiency. It would be good for the authors to explore.
4. The manuscript is well organized with the core ideas clearly described. But the presentation could be improved - there are a lot of very long sentences unnecessarily connected by “and”, “while”, etc. 
5. The legend for Fig 1A says “The first (DHS) generated by kallisto on ~2M DNase I sites … ”, but according to the figure and the main text, it should be“~3M”?

Is the rationale for developing the new method (or application) clearly explained?

Yes

Is the description of the method technically sound?

Yes

Are the conclusions about the method and its performance adequately supported by the findings presented in the article?

Yes

If any results are presented, are all the source data underlying the results available to ensure full reproducibility?

Yes

Are sufficient details provided to allow replication of the method development and its use by others?

Yes

Reviewer Expertise:

Bioinformatics, genomics, RNA structure, Genome structure, AI algorithms in biomedicine.

I confirm that I have read this submission and believe that I have an appropriate level of expertise to confirm that it is of an acceptable scientific standard, however I have significant reservations, as outlined above.

# Response

**Published:** 2020-05-20

**Authors:** Davide Cittaro

**Affiliations:** San Raffaele Hospital, Milan, Italy

Thank you for reviewing our manuscript and for the helpful comments. We have addressed major and minor points as detailed in the point-by-point response:

*The method was only tested with one dataset - PMBC. In fact, single cell ATAC-seq data is usually very sparse. The PMBC dataset used in this study is of relatively high quality. The method remains to be tested on more datasets, especially on those of more sparse, lower-quality.*

We agree that the PBMC dataset is of high quality. It was used as it could be considered a *de facto* standard in single cell analysis as it includes several populations at different degrees of separations ( *i.e. *B-cells and T-Cells are well separated, while NK and CD8 are less clearly distinguished). We also would like to point out that it is difficult to identify low quality scATAC-seq datasets for two reasons: one is the relative novelty of this technique and the other is the positive bias in publications, which generally lack of negative or low-quality results. Nevertheless, we tried to address this question analyzing data for K562 cell line. Cell lines are supposedly more homogeneous, data were obtained on a low-throughput platform (Fluidigm C1). We believe that it could be considered a good example of “lower quality” dataset, compared to the PBMC, at least considering the information content. We show that our strategy is consistent with standard approaches based on alignment and peak identification, we can identify the same level of residual heterogeneity.

*The key advantage of the method is presumably the much improved computational efficiency – there may be other advantages brought by the reference-based method. However, there is no results/statistics on the running time and memory usage in the manuscript. From the description, the improvement should be dramatic. I think it would be very nice to include a table or a figure to demonstrate the increase of computational efficiency. This could be a very helpful way to convince potential users.*

Thank you for this comment. We benchmarked *kallisto+bustools *and compared it to *cellranger-atac*, the default application for 10x data. We added a dedicated section in the main text, which shows the large reduction in required resources. Note that the *cellranger-atac* pipeline includes several steps that are common in downstream analysis (such as Seurat or Scanpy).  In order to make it fair, as explained in the text, we did not consider these steps in the comparison. In addition, we added runtime analysis for the approaches used in the analysis of K562 data.

*As in the above, this whole strategy is so different. It is thus possible for the method to be used for some other scATAC-seq data analysis with advantages not only in computational efficiency. It would be good for the authors to explore.*

Our work has been mainly motivated by the reduced resources that are needed by a kallisto-based approach, as we predict the number of scATAC-seq experiments will increase as well as the number of cells profiled. We anticipated additional advantages of a reference-based strategy in the first version of our manuscript, *e.g.* the availability of promoter-enhancer/gene interactions which could be readily applied to scATAC-seq data. During the revision process we had the opportunity to perform the analysis with non-optimal conditions ( *i.e.* peak identification from single end reads instead of paired end), which led to slightly different results. This serendipitous finding suggests that our strategy, not relying on *de novo* identification, improves the stability of cell characterization and, therefore, the reproducibility of results. We added these observations in the discussion. 

Of course, the usage of standardized reference could pave the way to a new class of processing steps not currently performed. As an example, one could identify a set of regions known to be generally accessible (or not) to perform standardized QC. Another example could be the identification of regions that could be used to score the cell cycle phases in scATAC-seq data, much like what is normally done with scRNA-seq data. We feel that all these examples require a deeper analysis, which is beyond the scope of this work, and any undemonstrated procedure would be, at best, greatly speculative. Our aim was to show general consistence between diverse approaches, which we believe has been demonstrated.

*The manuscript is well organized with the core ideas clearly described. But the presentation could be improved - there are a lot of very long sentences unnecessarily connected by “and”, “while”, etc.*

Thank you for this comment, we modified the text to increase readability. 

*The legend for Fig 1A says “The first (DHS) generated by kallisto on ~2M DNase I sites … ”, but according to the figure and the main text, it should be“~3M”?*

Thank you for spotting the typo in the figure legend. We corrected accordingly.

# Peer review: Reviewer response for version 1

**DOI:** 10.5256/f1000research.25099.r61566

**Published:** 2020-03-30

**Authors:** Iros Barozzi

**Affiliations:** 1 Department of Surgery and Cancer, Imperial College London, London, UK

In their paper “Fast analysis of scATAC-seq data using a predefined set of genomic regions” Giansanti *et al.* suggest an efficient strategy to analyse scATAC-seq data using *kallisto* and *bustools*.

 The paper is clearly written, the proposed strategy is well conceived and tested, and it will be useful for many researchers in the field of regulatory genomics. Clear advantages of this strategy are the reduced requirements in terms of computational resources and shorter execution times, when compared to other pipelines such as *cellranger-atac*. This comes at a cost, most notably the chance of missing signals at regions that are not present in the reference set. Nevertheless, in my opinion evaluations about this being a limitation has to be made on a case-by-case basis, and the authors clearly pointed this out (among other limitations) in the discussion. The authors also provide access to the full code, datasets and documentation to reproduce the analyses.

 A wide range of parameters was tested, both in terms of handling and modifying the input sequences to make them suitable for *kallisto*, and in terms of pre- vs post- processing the genomic partition considered for indexing. Combinations that return results that are highly concordant with those obtained with *cellranger-atac* were highlighted. The authors then demonstrated the robustness of the biological inferences made using their strategy by showing a very large overlap with the results achieved by *cellranger-atac* (in terms of different groups of regions marking distinct clusters and clusters annotation based on label transferring from scRNA-seq data).

 I am wondering if a natural application of this strategy would simplify the characterization of chromatin state at highly repetitive regions of mammalian genomes (e.g. indexing a database of transposable elements). This task would otherwise be quite difficult to handle explicitly with pipelines such as *cellranger-atac*.

 I only have two minor comments:

- Can the authors provide more details about the analysis described in the paragraph “Identification of marker regions”? How were the cell groups defined? How were the top 1,000 peaks for each group selected/identified?
- Fig. 1C: description of the blue curve seems to be missing.

Is the rationale for developing the new method (or application) clearly explained?

Yes

Is the description of the method technically sound?

Yes

Are the conclusions about the method and its performance adequately supported by the findings presented in the article?

Yes

If any results are presented, are all the source data underlying the results available to ensure full reproducibility?

Yes

Are sufficient details provided to allow replication of the method development and its use by others?

Yes

Reviewer Expertise:

Genomics; Transcriptional Regulation; Single-cell Transcriptomics.

I confirm that I have read this submission and believe that I have an appropriate level of expertise to confirm that it is of an acceptable scientific standard.

# Response

**Published:** 2020-05-20

**Authors:** Davide Cittaro

**Affiliations:** San Raffaele Hospital, Milan, Italy

Thank you for reviewing our work and for the comments. We have addressed your minor concerns as follows.

*Can the authors provide more details about the analysis described in the paragraph “Identification of marker regions”? How were the cell groups defined? How were the top 1,000 peaks for each group selected/identified?*

We apologize for lack of clarity in the manuscript. Cell groups were identified with the Leiden method, while markers were identified with Wilcoxon rank-sum test. The complete list of instructions used in the analysis is part of the repository linked in the main text, nevertheless we modified the text adding these specific details.

*Fig. 1C: description of the blue curve seems to be missing.*

Thank you for pointing this out. The blue line represented the fit of the DHS data. We acknowledge colouring scheme was not appropriate and, moreover, the fit DHS500 data was missing. In the revised manuscript we modified the figure accordingly.
